# Assessment of Serum Vitamin D Status and Its Associated Health Problem Among Children With Protein Energy Malnutrition: A Cross-Sectional Research Study in Central Rural India

**DOI:** 10.7759/cureus.37859

**Published:** 2023-04-20

**Authors:** Pallavi Dhulse, Archana Maurya, Mayur B Wanjari

**Affiliations:** 1 Department of Child Health Nursing, Shrimati Radhikabai Meghe Memorial College of Nursing, Datta Meghe Institute of Higher Education & Research, Wardha, IND; 2 Department of Research and Development, Jawaharlal Nehru Medical College, Datta Meghe Institute of Higher Education and Research, Wardha, IND

**Keywords:** vitamin d, children, developmental delay, pain, protein energy malnutrition

## Abstract

Background

Protein energy malnutrition (PEM) is a condition that results from inadequate intake of both macronutrients and micronutrients, leading to a lack of energy. The condition can manifest quickly or gradually, ranging from mild to severe. It predominantly affects children in low-income countries who do not receive enough calories and proteins. In developed nations, it is more prevalent among older individuals. PEM is more common in children as they consume less protein. In rare cases in developed countries, it may result from fad diets or a lack of knowledge about children's nutritional requirements, especially in cases of milk allergy. Vitamin D plays a critical role in bone growth and development by facilitating the absorption of calcium and phosphorus from food and supplements. Additionally, vitamin D has been linked to a reduced risk of infections, immune system disorders, diabetes, high blood pressure, and heart disease.

Aims and objectives

The primary objective of this study is to evaluate serum vitamin D levels and their relationship with health complications in children affected by PEM. The specific aim is to estimate the serum vitamin D levels in children with PEM who exhibit symptoms of being underweight, stunting (limited linear growth), wasting (abrupt weight loss), or edematous malnutrition (kwashiorkor). Additionally, this study seeks to analyze the correlation between serum vitamin D levels and the associated health problems in children with PEM.

Materials and methods

This cross-sectional study employed an analytical research approach. A total of 45 children with PEM participated in the study. Data were collected through a venipuncture method, and serum vitamin D level was assessed using an enhanced chemiluminescence method. The children's pain was assessed using a visual analogue scale, and developmental delay was evaluated using an assessment chart. Data were analyzed using SPSS Version 22 (IBM Corp., Armonk, NY).

Results

The study's results indicate that a considerable proportion of children, specifically 46.6%, were deficient in vitamin D, whereas 42.2% exhibited insufficiency and only 11.2% had sufficient levels of the vitamin. Pain assessment using the visual analogue scale classification revealed that 15.6% of children reported no pain, 60% reported mild pain, and 24.4% reported moderate pain. The mean and standard deviation of vitamin D levels correlated with developmental delay were 4.22±0.212 and 5.34±0.438, respectively. Similarly, the mean and standard deviation of vitamin D levels correlated with pain were 4.22±0.212 and 2.98±0.489, respectively. The Pearson correlation coefficient for vitamin D levels and pain was 0.010, with a significant level of 0.989, significantly lower than the tabulated value at a 5% significance level.

Conclusion

Based on the study's findings, it was concluded that children who suffer from PEM are at a risk of developing vitamin D deficiency, which can result in adverse health outcomes, including developmental delay and pain.

## Introduction

Protein energy malnutrition (PEM) is a condition characterized by a lack of energy due to insufficient macronutrients and micronutrients. The onset of PEM can be sudden or gradual [1]. The severity of this condition can range from mild to moderate or severe. It mainly affects children in poor countries who do not receive enough calories and proteins in their diet. In contrast, older adults in developed countries are also susceptible to this condition [2].

PEM is a term used to describe various biological conditions that arise from a lack of food. Although the name suggests a protein deficiency, individuals affected by this condition may not necessarily lack protein but rather all forms of energy [3,4]. Proteins derived from food that is typically used for tissue growth or repair can also be used as a source of energy. PEM is rare in developed countries and is typically associated with malnourished individuals who have been neglected as children or are socially isolated. The severity and clinical signs of PEM indicate food deficits in marasmus and kwashiorkor. When an individual's susceptibility to infectious diseases increases, which would not typically be fatal, protein-energy deficiency can become a life-threatening condition [5,6].

Malnutrition is a condition that occurs when there is an imbalance between the supply of nutrients and energy and the body's requirement for them to support development, maintenance, and proper functioning, as described by the World Health Organization (WHO) [7]. PEM comprises a series of interconnected conditions such as marasmus, kwashiorkor, and intermediate phases of marasmus-kwashiorkor. Children with marasmus suffer from extreme wasting, whereas those with kwashiorkor experience nutritional edema and metabolic abnormalities such as hypoalbuminemia and hepatic steatosis. Although marasmus is considered an appropriate response to fasting, kwashiorkor is regarded as a maladaptive reaction, according to studies [8,9].

Vitamin D insufficiency is a prevalent public health issue globally. One billion people worldwide are estimated to be deficient in vitamin D [10]. Vitamin D is well-known for its role in supporting strong bones. Although the skin creates most of the vitamin D when exposed to sunlight, it is also present in small amounts in oily fish, meat, eggs, and vitamin D supplements [11]. Adequate exposure to ultraviolet (UV) light is essential to maintain levels of vitamin D. It has been established that a vitamin D deficiency can cause changes in the immune response, increasing the risk of infection. These diseases are believed to elevate the risk of vitamin D deficiency, especially if there is an increase in diarrhea and vomiting, fever, earache, or discharge. Fat-soluble vitamin D is crucial in calcium homeostasis and bone metabolism [12,13].

## Materials and methods

Study setting and design

This experimental research project was conducted at selected hospitals in Wardha city, Maharashtra, India. The study was carried out in October 2022.

Inclusion and exclusion criteria

The study included all parents who were willing to participate in this study and their children who were available at the time of data collection. Children with mild, moderate, and severe degrees of protein PEM were included. Children who were admitted to the critical care unit and mentally retarded children were excluded.

Variables

The independent variable was serum vitamin D status, and the dependent variables were associated health problems and PEM.

Sampling and sample size

We recruited 45 study participants using a purposive sampling technique. The sample size was calculated using the Cochran formula for sample size:

n = Z²α/2 × P(1-P) / E²

Where: Zα/2 = level of significance at 5%, i.e., 95% confidence interval = 1.96 P = prevalence rate of malnutrition in India = 10.18% = 0.1018 E = desired error of margin = 10% = 0.10.

N = 1.962 × 0.1018 × (1-0.1018) / 0.102 = 35.12

n = 45 samples included in the study.

Data collection tools

This study's data collection process involved using several tools like a questionnaire and physical assessment, including a structural response sheet for demographic data, blood samples collected through the venipuncture method, and assessing the children's health problems related to vitamin D deficiency. In this study, the assessment of pain was conducted using the visual analogue pain scale. The scale's composition in terms of the number of images or symbols utilized is known to vary, with common ranges including 3 to 5, 7, or 10 points. To illustrate, a 3-point visual pain scale may incorporate images that symbolize the absence of pain, mild pain, and severe pain. In contrast, a 10-point scale could feature images corresponding to varying degrees of pain, ranging from none to excruciating levels.

Data collection process

A total of 45 children aged 1-10 years with PEM were included in this study. The study participants were given information about the study and time to ask questions before the study began, and informed consent was taken from parents before they were included. After obtaining informed written consent, blood samples were collected from the children for investigation purposes. The data collection process was completed within the designated time frame, and the investigator thanked all participants and authorities for their cooperation.

Consent and ethical approval

The institutional ethics committee approved the study, conducted by the moral standards established for human research. All participant's parents provided informed written consent, and the study was assigned the reference number DMIMS(DU)/IEC/2021/290.

Statistical analysis

The data obtained from the study participants were entered into a Microsoft Office Excel spreadsheet and analyzed using SPSS Version 22 (IBM Corp., Armonk, NY). The qualitative data were characterized by utilizing percentages, while the chi-square test and student unpaired t-test were employed to establish the p-value.

## Results

This section presents the percentage-wise distribution of participants based on their demographic characteristics. The sample was characterized based on information collected on variables such as age, gender, degree of PEM, educational status of parents, nutritional pattern, and family monthly income; the results are presented in (Table 1).

**Table 1:** Percentage wise distribution of children according to their demographic characteristics

Demographic variable	Frequency	Percentage (%)
Age		
1-2 years	20	44.4%
3-4 years	15	33.3%
5> years	10	22.3%
Gender		
Male	17	37.8%
Female	28	62.2%
Degree of PEM		
Mild	5	11.1%
Moderate	16	35.6%
Sever	24	53.3%
Nutritional pattern		
Vegetarian	16	35.6%
Non-vegetarian	29	64.4%
Education of parents		
Illiterate	0	0
Primary	23	51.1%
Secondary	13	28.9%
Higher secondary	5	11.1%
Graduate	4	8.9%
Postgraduate or more	0	0
Family monthly income		
Below 5,000	11	24.4%
50,001-10,000	21	46.7%
10,001-15,000	12	26.7%
Above 15,000	1	2.2%

Estimation of serum vitamin D status revealed that out of 45 participants, 21 (46.6%) were deficient in vitamin D, 19 (42.2%) were insufficient, and 5 (11.2%) had sufficient levels of vitamin D (Table 2).

**Table 2:** Estimate serum vitamin D status

Estimate serum vitamin D status		Frequency	Percentage	Mean±SD
Level	Range			
Deficient	<20 ng/mL	21	46.6%	4.22±0.212
Insufficient	20-29 ng/mL	19	42.2%	
Sufficient	30-100 ng/mL	5	11.2%	
Potential toxicity	>100 ng/mL	0	0	

Assessment of pain revealed that 7 (15.6%) children reported no pain, 27 (60%) reported mild pain, and 11 (24.4%) reported moderate pain among children with PEM (Table 3).

**Table 3:** Associated of health problem (pain) among children with protein energy malnutrition

Assessment of pain		Frequency	Percentage	Mean±SD
Level	Range			
No pain	0	7	15.6%	2.98±0.489
Mild	1-3	27	60%	
Moderate	4-6	11	24.4%	
Severe	7-9	0	0	
Very severe	10	0	0	

Assessment of Developmental Delay: Among children aged one to two years, 10 exhibited gross motor delays, 12 had language delays, one had social delays, four experienced vision delays, and one exhibited self-care delays. Among children aged three to four years, six exhibited gross motor delays, one had fine motor delays, 16 had language delays, 18 experienced social delays, and three exhibited vision delays. Among the participants aged five years or older, eight exhibited gross motor delay, two had a fine motor delay, twelve displayed social delay, one had vision delay, and six showed self-care delay, as shown in Table 4.

**Table 4:** Associated health problem (developmental delay) among children with protein energy malnutrition

Developmental delay by age and type	Frequency	Percentage (%)	Mean±SD
Gross motor			5.34±0.438
1-2 years	20	44.4%	
3-4 years	15	33.3%	
5> years	10	22.3%	
Fine motor			
No	21	46.7%	
Yes	24	53.3%	
Language			
No	9	20.0%	
Yes	36	80.0%	
Social			
No	30	66.7%	
Yes	15	33.3%	
Vision/hearing			
No	21	46.7%	
Yes	24	53.3%	
Self-care			
No	27	60.0%	
Yes	18	40.0%	

The mean and standard deviation of developmental delay were 4.22±0.212 and 5.34±0.438, respectively, while the Pearson correlation coefficients for vitamin D and developmental delay were 0.019. The significant level was 0.02, lower than the tabulated value at a 5% significance level. Therefore, it can be concluded that there is a statistically significant association between serum vitamin D and developmental delay (Table 5).

**Table 5:** Correlation between assessment of pain with vitamin D df, degree of freedom; NS, non-significant

Vitamin D					Chi-square value	df	P-value
Pain		Deficient	Insufficient	Sufficient			
No pain	Frequency	4	2	1	5.616	4	0.230, NS
	%	21.1%	9.5%	20.0%			
Mild pain	Frequency	12	14	1			
	%	63.2%	66.7%	20.0%			
Moderate pain	Frequency	3	5	3			
	%	15.7%	23.8%	60.0%			

The mean and standard deviation of pain were 4.22±0.212 and 2.98±0.489, respectively, and their correlation with vitamin D was assessed using Pearson correlation coefficients. The results showed a correlation coefficient of 0.010 and a significant level of 0.989, much lower than the tabulated value at the 5% significance level. Therefore, it can be inferred that a statistically significant association exists between pain and serum vitamin D levels, as shown in Table 6.

**Table 6:** Correlation between assessment of developmental delay with vitamin D df, degree of freedom; S, significant; NS, non-significant

Developmental parameter			Vitamin D			Chi-square value	Df	P-value
			Deficiency	Insufficiency	Sufficiency			
Gross motor	No	Frequency	17	18	2	6.957	2	0.031, S
		%	89.5%	85.7%	40.0%			
	Yes	Frequency	2	3	3			
		%	10.5%	14.3%	60.0%			
Fine motor	No	Frequency	9	10	2	0.101	2	0.951, NS
		%	47.4%	47.6%	40.0%			
	Yes	Frequency	10	11	3			
		%	52.6%	52.4%	60.0%			
Language	No	Frequency	6	1	2	5.890	2	0.053, NS
		%	31.6%	4.8%	40.0%			
	Yes	Frequency	13	20	3			
		%	68.4%	95.2%	60.0%			
Social	No	Frequency	12	16	2	2.562	2	0.278, NS
		%	63.2%	76.2%	40.0%			
	Yes	Frequency	7	5	3			
		%	36.8%	23.8%	60.0%			
Vision/hearing	No	Frequency	9	11	1	1.708	2	0.426, NS
		%	47.4%	52.4%	20.0%			
	Yes	Frequency	10	10	4			
		%	52.6%	47.6%	80.0%			
Self-care	No	Frequency	13	10	4	2.736	2	0.255, NS
		%	68.4%	47.6%	80.0%			
	Yes	Frequency	6	11	1			
		%	31.6%	52.4%	20.0%			

Table 7 shows the correlation between serum vitamin D status and its associated health problem, indicating a significant correlation between vitamin D and developmental delay (p = 0.002), while the correlation between vitamin D and pain is insignificant (p = 0.010) (Table 7).

**Table 7:** Correlation between serum vitamin D status and its associated health problem among children with protein energy malnutrition S, significant

Vitamin D/health problem	Mean	SD	Pearson correlation	P-value
Vitamin D	4.22	0.212	0.010	0.010 S
Pain	2.98	0.489		
Vitamin D	4.22	0.212	0.019	0.002 S
Developmental delay	5.34	0.438		

The results show a significant association between demographic variables and vitamin D levels. Chi-square calculations revealed significant associations (p < 0.05) of vitamin D levels with age (χ2 = 0.691), gender (χ2 = 1.848), degree of PEM (χ2 = 2.726), nutritional status (χ2 = 1.148), education of parents (χ2 = 7.185), and family monthly income (χ2 = 12.01), all of which exceeded the table value. Age and degree of PEM were found to be significantly associated with vitamin D levels (p = 0.024 and p = 0.043, respectively), with highly significant results (p < 0.05) (Table 8).

**Table 8:** Association with the demographic variables with serum vitamin D status df, degree of freedom; S, significant; NS, non-significant

Demographic variable	Frequency	Deficient	Insufficient	Sufficient	Potential toxicity	Chi-square value	Df	p-value
Age						0.69	4	0.024, S
1-2 years	20	9	8	3	0			
3-4 years	15	7	7	1	0			
5> years	10	5	4	1	0			
Gender						1.84	2	0.397, NS
Male	17	10	6	1	0			
Female	28	11	13	4	0			
Degree of PEM						2.72	4	0.043, S
Mild	5	3	2	0	0			
Moderate	16	8	5	3	0			
Sever	24	10	12	2	0			
Nutritional pattern						1.148	2	0.563, NS
Vegetarian	16	9	6	1	0			
Non-vegetarian	29	12	13	4	0			
Education of parent						7.185	6	0.304, NS
Illiterate	0	0	0	0	0			
Primary	23	12	9	2	0			
Secondary	13	6	5	2	0			
Higher secondary	5	3	1	1	0			
Graduate	4	0	4	0	0			
Postgraduate or more	0	0	0	0	0			
Family monthly income						12.01	6	0.062, NS
Below 5,000	11	4	7	0	0			
50,001-10,000	21	12	7	2	0			
10,001-15,000	12	5	5	2	0			
Above 15,000	1	0	0	1	0			

## Discussion

The present study aimed to investigate the correlation between serum vitamin D and developmental delay. The mean and standard deviation for developmental delay were 4.22±0.212 and 5.34±0.438, respectively. The Pearson correlation coefficient was 0.019, and the significant level was 0.02, much lower than the tabulated value at a 5% significance level. Therefore, it can be interpreted that there is a statistically significant association between developmental delay and serum vitamin D. This finding is supported by a cross-sectional study conducted by Tavakolizadeh et al., [14]. The study investigated the relationship between Vitamin D deficiency and gross motor development in children, including 186 children. Of the 186 children, 92 were boys (49.5%). Among them, 40 (21.5%) patients could stand alone and sit without assistance, while 24 (12.9%) patients could only sit without assistance. Additionally, 122 (65.6%) patients could stand, sit, and walk without assistance. Vitamin D levels were sufficient in 148 (79.5%) children, too low in 32 (17.2%) children, and deficient in six (3.2%) children. Walking ability was significantly associated with sufficient vitamin D levels (p < 0.001, OR=3.9, 95% CI=1.9-8.4). The researchers concluded that children with gross motor developmental delay need to be thoroughly evaluated for vitamin D deficiency, given the significant correlation between vitamin D deficiency and gross motor developmental milestones in this population of children [14].

The correlation between vitamin D and pain was assessed in the present study, and the mean and standard deviation for pain were found to be 4.22±0.212 and 2.98±0.489, respectively. The Pearson correlation coefficient was 0.010, and the significant level was 0.989, much lower than the tabulated value at a 5% significance level. Therefore, it can be interpreted that pain is statistically associated with serum vitamin D. The present study is supported by the findings of Adegoke et al., [15] who conducted a cross-sectional study on the influence of serum 25-hydroxyvitamin D on the rate of pain episodes in Nigerian children. A total of 123 children were enrolled in the study using consecutive sampling. According to their findings, 123 children with sickle cell disease (HbSS) had an average (SD) blood 25-OHD level of 105.8 (24.1) nmol/L (range: 37.5-155.8). The levels in 14 (11.4%) patients were either inadequate (1.6%) or insufficient (9.8%). None of the patients exhibited significant vitamin D deficiency. The results of the study revealed that pain was experienced by all children with low vitamin D levels and 69.7% of children with normal vitamin D levels (14 [100%] vs. 76 [69.7%], 95% CI: 0.7-0.9, p = 0.04]. Compared to the 33 patients with no pain episodes, the mean serum vitamin D level in the 90 patients with at least one pain episode was 103.1 (25.2) nmol/L (95% CI: 1.3-7.8, p = 0.04), which was significantly lower. The level of serum 25-OHD was inversely linked with pain frequency. Significant pain episodes were predicted by vitamin D serum levels (OR: 1.2, 95% CI 1.3-1.7, p = 0.04) and fetal hemoglobin concentration (OR: 1.6, 95% CI: 1.1-1.4, p = 0.02). The researchers found a correlation between the elevated frequency of acute pain episodes in children and low serum vitamin D levels [15].

The present study assessed the association between demographic variables and vitamin D levels using chi-square calculations, and significant associations were observed at p < 0.05 for several variables. These included age (χ2 = 0.691), gender (χ2 = 1.848), degree of PEM (χ2 = 2.726), nutritional status (χ2 = 1.148), education of parents (χ2 = 7.185), and family monthly income (χ2 = 12.01), all of which were found to be greater than the table value. The results also revealed a significant association between age and degree of PEM (p = 0.024, p = 0.043, respectively), indicating a highly significant p < 0.05. Kumari conducted a cross-sectional study on the prevalence of PEM among children under five in rural areas of Ambala, Haryana, India, which supports our findings [16]. A total of 300 children were examined using nonprobability consecutive sampling, and descriptive statistics and inferential analysis were employed for data analysis. According to the Gomez categorization, the results showed that 44.43% of the children had good nutritional status, with the proportion of first-, second-, and third-degree malnutrition being 39.34%, 15.66%, and 0.66%, respectively. The researchers concluded that the children's ages significantly influenced the association between PEM and PEM [16].

Limitations of the study

The sample size is relatively small, which could limit the generalizability of the findings to larger populations. Additionally, the study relies on self-reported participant data, which could introduce bias and inaccuracies. Furthermore, the study design is cross-sectional, which limits the ability to conclude causality. Finally, the study was conducted in a specific geographic location and may not represent other regions or cultures. Despite these limitations, the study provides valuable insights into the topic of interest and lays a foundation for future research.

## Conclusions

Based on the current study's findings, a substantial correlation exists between demographic variables and vitamin D levels among children experiencing PEM in rural areas of central India. The investigation reveals that the age and severity of PEM play a significant role in this association. These findings' implications are significant in combating malnutrition and associated health issues among children in central rural India. By highlighting the importance of demographic variables in determining the vitamin D status of children with PEM, this study underscores the need for targeted interventions considering the age and the severity of malnutrition. Addressing these factors through targeted interventions and policies may help improve the overall health outcomes of children suffering from malnutrition in this region. Furthermore, these findings could have broader implications for similar populations and regions facing similar malnutrition and vitamin D deficiency challenges. These findings offer valuable insights for designing effective interventions and policies to mitigate the impact of malnutrition and associated health complications in rural areas of central India.
